# Outcomes after Treatment of Metaplastic Versus Other Breast Cancer Subtypes

**DOI:** 10.7150/jca.40817

**Published:** 2020-01-01

**Authors:** Amy C. Moreno, Yan Heather Lin, Isabelle Bedrosian, Yu Shen, Gildy V. Babiera, Simona F. Shaitelman

**Affiliations:** 1 Department of Radiation Oncology, The University of Texas MD Anderson Cancer Center, Houston, TX.; 2 Department of Biostatistics, The University of Texas MD Anderson Cancer Center, Houston, TX.; 3 Department of Surgical Oncology, The University of Texas MD Anderson Cancer Center, Houston, TX.

**Keywords:** metaplastic breast cancer, triple negative breast cancer, breast cancer outcomes, radiation therapy, chemotherapy.

## Abstract

**Purpose**: Metaplastic breast cancer (BC) is an uncommon yet aggressive histologic subtype of BC. We sought to identify factors associated with its diagnosis and compare the management and outcomes of metaplastic BC with those of other BCs and triple negative invasive ductal carcinoma in particular given how often it has a triple negative phenotype.

**Patients and Methods**: We identified women diagnosed with invasive BC in 2010-2014 in the National Cancer Data Base, and used univariate analysis to compare baseline patient and tumor characteristics by BC subtype. Overall survival (OS) was estimated with the Kaplan-Meier method, and multivariate Cox proportional hazards models were used to identify independent predictors of OS.

**Results**: Of 247,355 cases, 2,084 (0.8%) were metaplastic BC, 55,998 (23%) triple negative BC, and 77% other BC. Relative to non-metaplastic BC, women with metaplastic BC were more likely to be older at diagnosis (median age, 62 vs. 59 years), have ≥1 comorbid conditions (22% vs. 18%), and be on Medicare (41% vs. 33%; *P*<0.001). Metaplastic BCs tended to be basal-like (77%), and relative to triple-negative or other BC, metaplastic BC was associated with higher clinical T status (cT3-4, 18% vs. 11%, 8%), no clinical nodal involvement (cN0, 86%, 77%, 80%), no lymphovascular invasion (72%, 65%, 62%), and high-grade tumors (71%, 77%, 35%) (*P*<0.001). Most metaplastic BCs were treated with mastectomy (58%), sentinel lymph node dissection (65%), chest wall or breast irradiation (74%), and chemotherapy (75%) as adjuvant therapy (60%). At a median follow-up time of 44.5 months, OS rates were lower for metaplastic BC than for triple-negative or other BC across all clinical stages at 5 years (stage I, 85%, 87%, 91%; II, 73%, 77%, 87%; III, 43%, 53%, 75%) and at 3 years (Stage IV, 15%, 22%, 64%; *P*<0*.*001). On multivariate analysis, increasing age, advanced clinical stage, lymphovascular invasion, axillary (vs. sentinel) node dissection, and no radiation or chemotherapy were associated with worse outcomes in metaplastic BC. Extent of surgery affected survival for triple-negative and other BC but not for metaplastic BC.

**Conclusion**: Outcomes for metaplastic BC continue to be worse than those for other BC subtypes despite modern treatments. Optimizing systemic therapy options, which was a significant predictor of survival, should be a priority in managing metaplastic BC.

## Introduction

Although breast cancer is the most common cancer diagnosis affecting women, with more than 268,000 cases documented annually, metaplastic breast cancer (BC) constitutes less than 1%-2% of all breast cancer cases.^1^,^2^ Clinically, metaplastic BC manifests as a rapidly growing breast mass with complex echogenicity (i.e., solid and cystic components) on ultrasonography and a high-density mass on mammography.^3^ Histologically, metaplastic BC is composed of a heterogeneous population of tumors that can be classified by the presence of non-glandular or mesenchymal cell types or by further categorization into subtypes depending on the presence of various features such as osteoclastic giant cells or spindle cells^4^-^7^.

At presentation, the rate of clinical lymph node involvement in metaplastic BC is typically low. However, metaplastic BC is more often diagnosed at advanced clinical stages due to larger primary tumors compared with other BCs and is associated with higher rates of chemoresistance, distant metastasis, and worse overall survival (OS).^4^,^8^ Metaplastic BC has a particularly high prevalence of triple-negative receptor status or the absence of estrogen receptor, progesterone receptor, and human epidermal growth factor receptor 2 (HER2) amplification.^9^ Without targetable proteins, metaplastic BC remains challenging to treat. Prospective data are limited for choosing the best treatment for metaplastic BC given the rarity of the diagnosis. Instead, much of the current standard of care for metaplastic BC has been extrapolated from findings of small single-institution series or case reports. We therefore used the National Cancer Data Base (NCDB), which captures approximately 70% of all diagnosed cancer cases in the United States, to evaluate the characteristics, management, and outcomes based on treatment and tumor features of patients with metaplastic BC compared to those with triple-negative BC and all other BC subtypes. We also used multivariate analysis to identify independent predictors of survival for women with metaplastic BC.

## Patient and methods

### Data Source and Cohort Selection

A joint project established in 1989 by the Commission on Cancer of the American College of Surgeons and the American Cancer Society, the NCDB has collected oncologic data from approximately 1,500 accredited facilities in the United States, totaling over 34 million records^10^. Available patient information is de-identified and therefore exempt from human protection oversight by the institutional review board.

**Figure 1:**
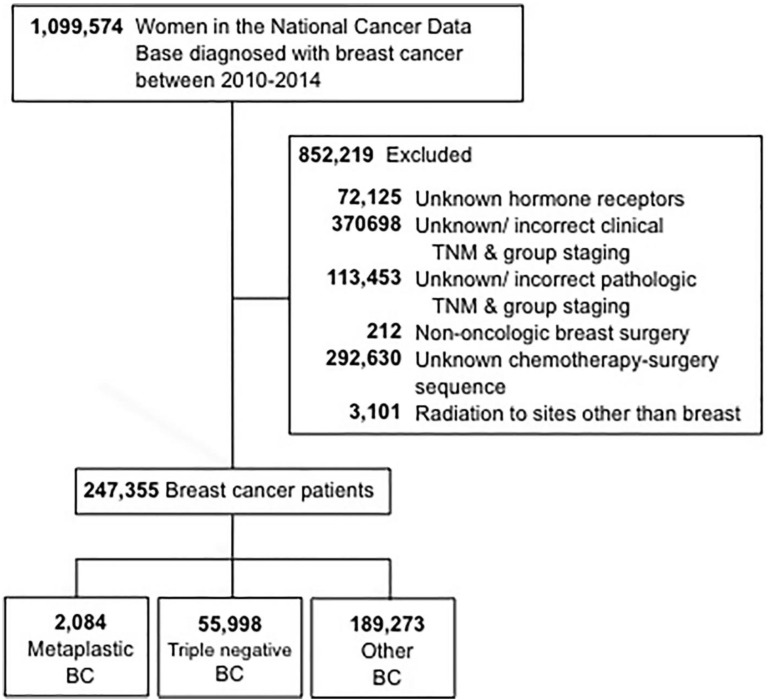
CONSORT diagram. BC, breast cancer.

The selection criteria used for this study are outlined in Figure 1. Women aged 18 and older who were diagnosed with invasive breast cancer from 2010 through 2014 were included. Patients with unknown hormone (estrogen and progesterone) receptor (HR) status were excluded. Additional reasons for exclusion were missing information regarding surgery, chemotherapy, or clinical staging according to the American Joint Committee On Cancer (AJCC) 6^th^ or 7^th^ edition. Patients were then stratified into three groups: 1-metaplastic BC (with the histology codes 8575 or 8573), 2- triple-negative BC (TNBC, which included only non-metaplastic histologies), and 3-all other remaining types of BC. The term “non-metaplastic BC” was utilized to refer to triple-negative BC and other BC combined.

### Variables

Information obtained and analyzed from the NCDB included patient age at diagnosis, year of diagnosis, race, Charlson/Deyo comorbidity index, medical insurance status, median household income, and treating facility type (dichotomized into academic/research versus non-academic). Clinicopathologic features included AJCC clinical T and N (nodal) designation, overall AJCC clinical stage group, HR status, HER2 receptor status, tumor grade, and lymphovascular invasion (LVSI). Molecular subtypes were defined as being HR(+)/HER2(-), HER2(+), triple-negative (estrogen receptor(-), progesterone receptor (-), HER2(-)), or unknown. Patients with metaplastic BC with triple-negative receptor status were labeled as tn-metaplastic BC to distinguish them from non-metaplastic TNBC. Primary management included surgery (either breast-conserving, mastectomy, or none), radiation therapy with or without inclusion of regional nodal irradiation, chemotherapy usage (neoadjuvant, adjuvant, or none), and hormone therapy. Surgical management of the axilla was defined as either an axillary node dissection (ALND), with 10 lymph nodes or more removed, or a sentinel lymph node dissection (SLND), when <10 lymph nodes were removed^11^,^12^.

### Statistical Analysis

Univariate analysis was used to evaluate potential associations between demographic, tumor and clinical characteristics and a diagnosis of metaplastic BC, using chi-square tests for categorical variables and *t* test/analysis of variance or their counterparts of the non-parametric approaches (Wilcoxon rank-sum or Kruskal-Wallis) for continuous variables^13^. OS was defined as from the time of diagnosis to the time of death. OS time for the surviving patients was right-censored at the time of last contact. The distribution of OS was estimated by the Kaplan-Meier method^14^. The log-rank test^15^ was used to test the difference in survival between groups. Regression analyses of survival data based on the Cox proportional hazards model^16^ were conducted on OS. A multivariate Cox proportional hazards model was obtained by first including an initial set of candidate predictor variables with a *P* value < 0.05 in the univariate analysis. Stepwise backward elimination was then used, with 0.05 for the significance level of the Wald chi-square for an effect to stay in the model.

Survival analyses were conducted within each of the cancer types separately.

Facility location and type were not included in the metaplastic BC model selection process because neither factor was statistically significant for this group in the univariate setting and data were not fully available for patients younger than 40 years. To explore if details of systemic therapy and radiation delivery affected outcomes among patients with metaplastic BC, we performed subset analyses on this cohort to evaluate the potential influence of chemotherapy sequencing and extent of radiation targets on OS. All tests were two-tailed, and statistical significance was defined as a *P* value <0.05. SAS version 9.4 (SAS Institute, Cary, NC) and S-Plus version 8.2 (TIBCO Software Inc., Palo Alto, CA) were used to carry out the computations for all analyses.

## Results

### Cohort and Tumor Characteristics

We identified 247,355 women with a diagnosis of invasive breast cancer in 2010-2014, of whom 0.8% (n=2,084) had metaplastic BC, 23% (n=55,998) TNBC, and 77% (n=189,273) had other BC. Patient sociodemographic, clinical, and pathologic tumor characteristics and treatment characteristics by BC type are shown in Table 1. The median age was 63 years (range 22-90) for women with metaplastic BC and 59 years (range 18-90) years for both TNBC and other BC. Women with metaplastic BC more commonly had a comorbidity score of ≥1 (22% vs. 18% TNBC vs. 16% other BC) and had public insurance (50% vs. 42% TNBC vs. 41% other BC) (all *P*<0.001). The diagnosis of metaplastic BC or TNBC was more commonly associated with black race than was other BC (18% vs. 21% vs. 11%, *P*<0.001).

Significant differences in clinical disease stage and tumor features were seen by cancer type. Metaplastic BC was often diagnosed at more advanced stages, with only 31% of patients with metaplastic BC having clinical stage I disease versus 46% of TNBC and 54% of other BC (*P*<0.001). This pattern seemed to correlate with higher rates of larger tumors in the metaplastic BC group (cT3-4, 18% vs. 11% vs. 8%, respectively), but the presence of clinical nodal involvement was the lowest for metaplastic BC (cN1-3, 14% vs. 23% vs. 20%, respectively) (all *P*<0.001). In contrast to the clinical nodal status, rates of pathologic nodal involvement was slightly lower for metaplastic BC and TNBC but doubled for other BC (pN1-3, 18% vs. 26% vs. 40%, respectively, *P*<0.001). With respect to receptor status, 77% of the metaplastic BC group had tn-metaplastic BC, which is 3.4 times higher than the non-metaplastic BC group (23%), of which HR(+)/HER2(-) was the most common molecular subtype at 59% (*P*<0.001). Moreover, only 5% of metaplastic BC was HER2(+) compared with 16% of non-metaplastic BC. Both metaplastic BC and TNBC had nearly twice the proportion of grade 3 tumors relative to other BC (71%, 77%, and 35%, respectively, *P*<0.001), and metaplastic BC had lower rates of LVSI relative to TNBC and other BC (13%, 20%, and 24%, respectively, *P*<0.001).

### Treatment by Breast Cancer Type

Most patients (99%) underwent oncologic breast surgery, including surgical evaluation of the axilla (97%). Mastectomy was more commonly used in the metaplastic BC group (58% vs. 47% TNBC vs. 51% other BC, *P*<0.001), as was SLND (65% vs. 62% TNBC vs. 58% other BC; *P*<0.001). Regional nodal irradiation was more often used as a part of treatment for other BC than for metaplastic BC and TNBC (26%, 27%, and 34%; *P*<0.001), respectively.

With regard to systemic therapy, neoadjuvant chemotherapy was used nearly twice as often for metaplastic BC and TNBC than for other BC (16%, 23%, 9%; *P*<0.001), and approximately one quarter of all women did not receive any chemotherapy. Hormone therapy was used the least often for TNBC (2%) but was part of the treatment paradigm for 11% of metaplastic BC and 55% of other BC cases.

### Survival Analysis

**Figure 2:**
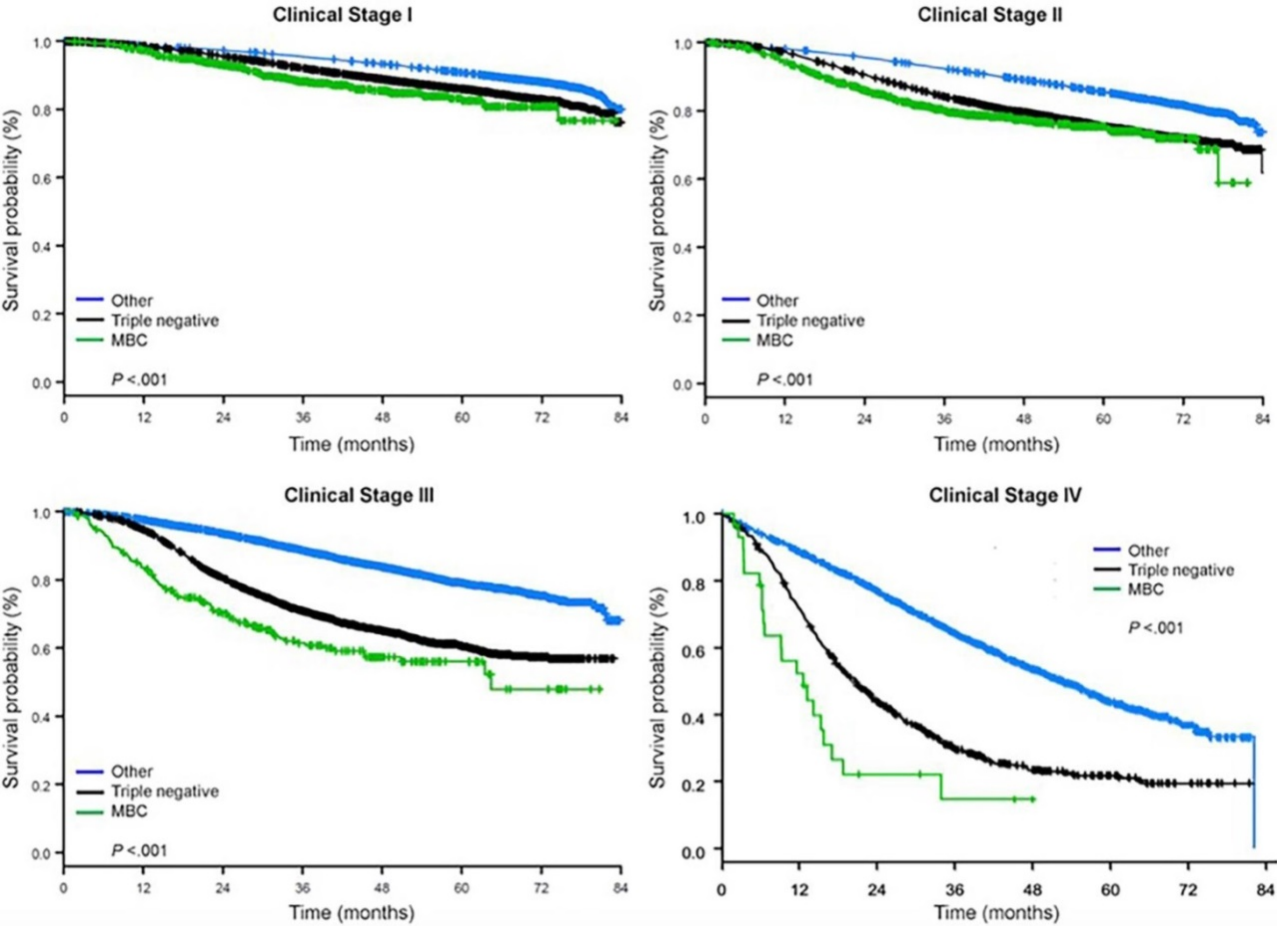
Overall survival curves of breast cancer types by clinical stage, examining metaplastic, triple negative, and other breast cancers. Abbreviation: MBC, metaplastic breast cancer.

**Figure 3:**
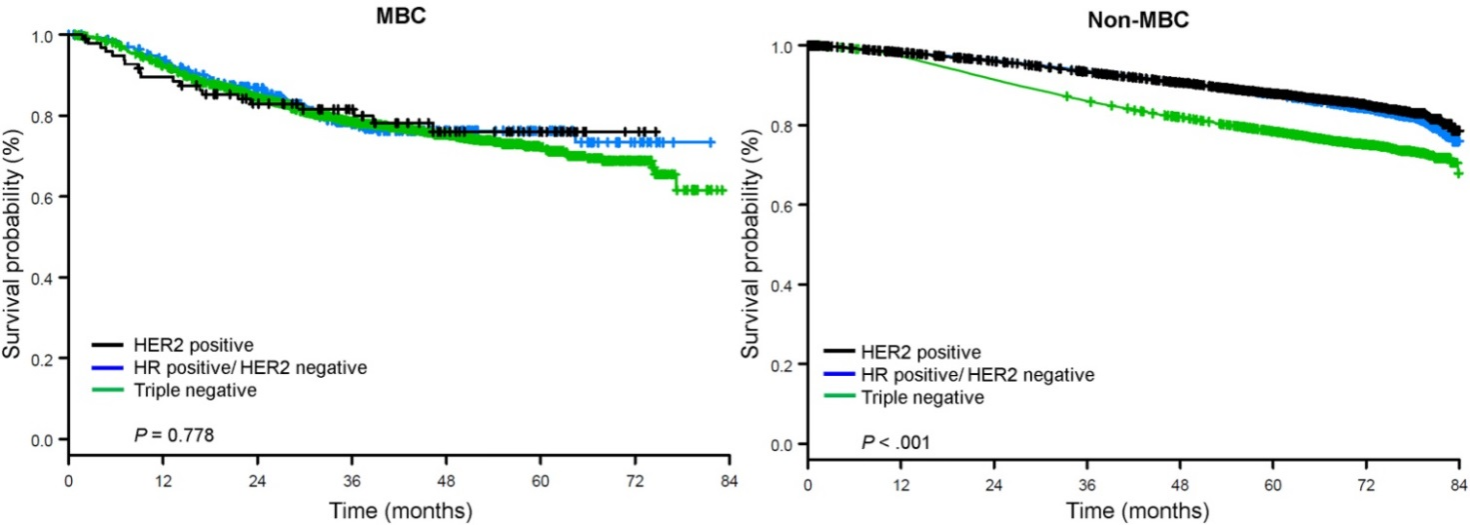
Overall survival curves for patients with metaplastic and non-metaplastic breast cancer stratified by receptor status. Abbreviations: HER2, Human epidermal growth factor receptor 2; HR, hormone receptor; MBC, metaplastic breast cancer.

**Figure 4:**
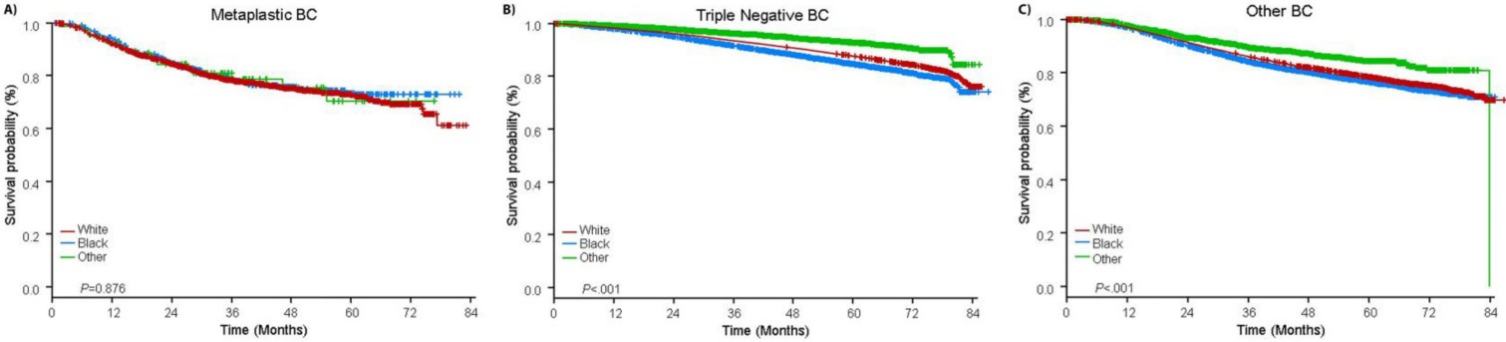
Overall survival curves for patients with metaplastic, triple negative, and other breast cancer stratified by race. Abbreviations: BC, breast cancer.

The median follow-up time was 44.5 months. Patients with metaplastic BC had significantly worse unadjusted OS regardless of clinical stage (Fig. 2). The 5-year OS estimates for the metaplastic BC, TNBC, and other BC patients with stage I disease were 85%, 87% and 91%; those for stage II were 73%, 77%, and 87%; and those for stage III were 43%, 53%, and 75% (*P*<0.001). The 3-year OS rates for patients with metastatic disease were 2-4 times higher for the TNBC and other BC groups at 30% and 64% compared with 15% for metaplastic BC (*P*<0.001). Survival by molecular subtype was also analyzed (Fig. 3). Among patients with metaplastic BC, no differences in OS were apparent by molecular subtype (HR(+)/HER2(-), HER2(+), or tn-metaplastic BC) (*P=*0.778). In contrast, for non-metaplastic BC, the triple-negative subtype was associated with worse OS than the HER+ and HR(+)/HER2(-) subtypes (*P*<0.001). Race also did not seem to be associated with survival among patients with metaplastic BC (5-year OS rates 73% for white vs. 74% for black, *P=*0.876), whereas race was associated with OS in both the TNBC (*P*<0*.*001) and other BC groups (*P*<0.001; Fig. 4).

On multivariate analysis, increasing age, higher clinical T classification, the presence of clinical nodal disease or LVSI, and treatment with ALND all correlated with worse outcomes for patients with metaplastic BC, TNBC, and other BC (Supplemental Table 1). The addition of chemotherapy and radiation therapy both independently improved OS regardless of breast cancer type. Other patient and tumor characteristics associated with survival in TNBC and other BC, including race, comorbidities, and tumor grade, were not associated with survival outcomes for metaplastic BC on multivariate analysis.

Given the relatively poor outcomes among patients with metaplastic BC, subgroup analyses were done on the metaplastic BC group to assess if any treatment modalities were associated with improved outcomes. After adjusting for nodal status (cN0 vs. N+), treatment with more aggressive axillary surgery (ALND vs. SLND [hazard ratio {HR} 1.3, 95% confidence interval {CI} 1.1-1.7, *P*=0.01] was associated with worse outcomes, a phenomenon which was also seen among TNBC and other BC (Table 2). Among those receiving radiation therapy, treatment with regional nodal irradiation vs. breast- or chest wall-only radiation did not significantly influence outcomes among patients with metaplastic BC (*P*=0.077 and 0.200 for clinical N0 and N+, respectively). Receipt of neoadjuvant chemotherapy was associated with worse outcomes than adjuvant chemotherapy among patients with clinically node-negative metaplastic BC (HR 1.9, 95% CI 1.3-2.6, *P*<0.001), but not for those with clinically node-positive metaplastic BC (*P*=0.43).

## Discussion

Metaplastic breast cancer is a rare yet particularly aggressive form of breast cancer, especially when compared against other breast tumor subtypes. This disease entity has been relatively under-represented in the literature. An extensive search by Rayson et al. of publications from 1966 to 1997 yielded a total of only 27 cases.^17^ Large national databases such as the NCDB are advantageous for studying rare cancers such as metaplastic BC.^18^ However, the incidence of metaplastic BC diagnosis in the United States according to these databases is still low at less than 500 cases per year.^9^,^19^ In our study, we found that metaplastic BC was most commonly diagnosed as a large tumor with adverse risk features such as poorly differentiated tumor grade and triple-negative receptor status. Patients with metaplastic BC had significantly worse survival regardless of stage at presentation compared with triple-negative and other BC. Receipt of chemotherapy and radiation therapy were independent predictors of better OS, but the sequencing of chemotherapy seemed to affect outcomes, particularly for patients with metaplastic BC with no clinical nodal involvement, acknowledging that some of these patients had pathologic nodal involvement. However, our findings may reflect potential heterogeneity in the underlying biological mechanisms driving responses to treatments in this particular group of BC patients and highlight the need for better risk stratification and systemic therapy options to improve outcomes.

Although most metaplastic BCs have a triple-negative phenotype, the behavior of metaplastic BC seems to be unique compared with other TNBCs. TNBC tends to have a worse prognosis than other types of BC,^20^-^23^ and the notion that metaplastic BC is more aggressive than TNBC has been corroborated by multiple smaller, retrospective investigations.^4^,^8^,^9^,^24^ Such studies have shown similar patterns of diagnosis at advanced stages for metaplastic BC versus TNBC, owing to higher rates of cT3-4 disease rather than nodal involvement,^9^,^24^ and in a single-institution review of 46 cases of metaplastic BC, patients with metaplastic BC had a significantly higher risk of local disease recurrence (30% vs. 15%; *P*=0.004). These features ultimately correlated with inferior 5-year disease-free survival rates (30% vs. 90%; *P*<0.001) and OS rates (65% vs. 87%; *P*=0.002) for patients with metaplastic BC relative to TNBC.^24^ A Surveillance Epidemiology and End Results study of 1,1112 patients with metaplastic BC also showed worse cancer-specific survival rates at 3 years for metaplastic BC than for TNBC (78% vs. 84%).^9^ When examining tn-metaplastic BC, Li et al. found worse disease-free survival (HR 1.48, 95% CI 1.19-1.84, *P*<0.01) and OS (HR 1.42 [1.17-1.73]; *P*<.01) for tn-metaplastic BC compared with TNBC. Our study, which included one of the largest groups of patients with metaplastic BC to date (n=2,084), reinforces these findings and highlights that receptor status is not an independent predictor of survival for metaplastic BC (*P*=0.778) as it is for non-metaplastic BC.

A critical therapeutic component associated with improved outcomes for all metaplastic BC patients in our study was the use of chemotherapy (HR 0.58, 95% CI 0.45-0.75, *P*<0.001). Our report is the first to our knowledge to report on outcomes in metaplastic BC based on sequencing of chemotherapy. Strikingly, our subanalysis found that for patients with cN0 metaplastic BC in particular, neoadjuvant chemotherapy was associated with worse OS compared with adjuvant chemotherapy (HR 1.88, 95% CI 1.34-2.64, *P*<0.001), whereas chemotherapy sequencing had no effect on clinically node-positive metaplastic BC. One possible explanation for this may be that our patients with metaplastic BC had higher clinical T status, which in other reports has been associated with lower rates of pathologic complete response and subsequent worse survival outcomes.^25^ Moreover, clinical staging methods (i.e. physical examination alone, incorporation of ultrasonography) can introduce heterogeneity in a clinical N0 population by missing true nodal disease burden, which in itself can be associated with inferior outcomes. Overall, metaplastic BC response to neoadjuvant chemotherapy has been reported to be as low as 18%,^17^ which is much lower than known response rates for TNBC to anthracycline- and taxane- based chemotherapy regimens^26^,^27^.

Based on the poorer survival and relatively rapid development of distant metastatic spread^28^ compared to other breast cancer subtypes noted in our study and in other reports, the need for better systemic therapy options is clearly evident for metaplastic BC. Transcriptional profiling has shown this cancer subtype to exhibit a tumorigenic signature with stem cell-like characteristics, frequent aberrations in the PI3K/AKT/mTOR pathway, and overexpression of vascular endothelial growth factor (VEGF).^29^ These characteristics are similar to those found in mesenchymal TNBCs.^30^,^31^ Promising efforts are underway to identify alternative systemic therapy regimens for these patients, including a recent phase I trial that showed improved objective response rates to liposomal doxorubicin, bevacizumab (monoclonal antibody to VEGF-A), and everolimus (mTOR inhibitor) in patients with metaplastic TNBC with a PI3K pathway aberration^32^,^33^.

With regards to local treatments, mastectomy was more commonly used in our study for metaplastic BC but was not associated with improved survival relative to breast-conserving surgery. Also, no survival benefit was found from treatment that incorporated ALND versus SLND. Radiation therapy, however, was a significant predictor of survival, with metaplastic BC patients treated with adjuvant radiation therapy being 30% less likely to die than those who did not receive radiation. Relevant limitations in our radiation therapy analysis include unknown details regarding treatment planning, selected modality, quality assurance, or whether techniques such as deep inspirational breath-hold were used.^34^,^35^ While local-regional relapse cannot be examined in the NCDB, an institutional series of 113 patients with metaplastic BC (54% which received radiation therapy) showed that radiation was the only factor which correlated with reduced locoregional recurrence (relative risk without radiation 3.1; 95% CI 1.13-9.88, *P*=0.027)^36^.

Our results should be interpreted with caution given the major limitations of not knowing which chemotherapy regimens were used, the duration of treatments, or response to neoadjuvant chemotherapy, as that information is unavailable in the NCDB. We also acknowledge the limitations of clincopathologic risk factors captured in the NCDB, without which it may not be feasible to fully characterize clinical differences that drive some of the outcome differences we have found, such as worse survival outcomes with ALND even when controlling for lymph node status. Although our sample size of metaplastic BC patients was small relative to non-metaplastic BC studies, it is paradoxically also likely the largest existing study to date examining this question given the limited numbers of patients with metaplastic BC reported in institutional series.^37^,^38^ Additional investigations are needed to understand the biological predilection for metastatic spread in metaplastic BC and validate our findings in separate datasets of metaplastic BC patients.

Our current standard of care is clearly not adequate for this unique cancer population, and obtaining randomized data on metaplastic BC in the future will require cooperative efforts owing to the small numbers of patients. The NCDB provides a noteworthy strength, which is the ability to study treatment patterns and outcomes associated with a rare diagnosis. With 2,084 individuals with metaplastic BC in this cohort, this is one of the largest metaplastic BC investigations reported in the modern era. Survival for these patients is poor relative to those with any other BC types, and although this fact likely prompts the incorporation of aggressive therapy, our study suggests that more extensive local-regional treatment (e.g., ALND or regional node irradiation) should be carefully considered on a case-by-case basis. Overall, the use of systemic therapy is crucial for the management of metaplastic BC regardless of nodal burden, and the development of effective targeted therapies based on tumor genomic profiling analysis shows promise for the future.

## Supplementary Material

Supplementary table.

## Figures and Tables

**Table 1:** Patient and treatment characteristics.

Characteristic	Metaplastic BC, No. (%)	Triple-Negative BC, No. (%)	Other BC, No. (%)	*P* Value
**Median age, years (range)**	62 (22-90)	59 (18-90)	59 (18-90)	<0.001
**Race**				
** White**	1623 (77.9)	41791 (74.6)	158406 (83.7)	<0.001
** Black**	368 (17.7)	11771 (21)	20722 (10.9)	
** Other**	80 (3.8)	2041 (3.6)	8507 (4.5)	
** Unknown**	13 (0.6)	395 (0.7)	1638 (0.9)	
**Comorbidity score**				
** 0**	1635 (78.5)	45882 (81.9)	158177 (83.6)	<0.001
** 1**	349 (16.7)	8107 (14.5)	25524 (13.5)	
** >=2**	100 (4.8)	2009 (3.6)	5572 (2.9)	
**Median income**				
** <$30,000**	251 (12)	7110 (12.7)	19154 (10.)	<0.001
** $30,000-$34,999**	332 (15.9)	9081 (16.2)	28472 (15)	
** $35,000-$45,999**	580 (27.8)	15084 (26.9)	48845 (25.8)	
** $46,000+**	858 (41.2)	23050 (41.2)	86637 (45.8)	
** Unknown**	63 (3)	1673 (3)	6165 (3.3)	
**Medical insurance**				
** Private**	975 (46.8)	30263 (54)	105839 (55.9)	<0.001
** Medicaid**	154 (7.4)	4716 (8.4)	13267 (7)	
** Medicare**	862 (41.4)	18355 (32.8)	61535 (32.5)	
** Other**	19 (0.9)	665 (1.2)	2212 (1.2)	
** Uninsured**	43 (2.1)	1422 (2.5)	4163 (2.2)	
** Unknown**	31 (1.5)	577 (1)	2257 (1.2)	
**County type**				
** Metropolitan**	1720 (82.5)	46252 (82.6)	157058 (83)	0.152
** Urban**	271 (13)	7456 (13.3)	24367 (12.9)	
** Rural**	43 (2.1)	951 (1.7)	3219 (1.7)	
** Unknown**	50 (2.4)	1339 (2.4)	4629 (2.4)	
**Facility type**				
** Academic**	668 (32.1)	16117 (28.8)	52120 (27.5)	<0.001
** Non-Academic**	1318 (63.2)	35472 (63.3)	126101 (66.6)	
** Unknown**	98 (4.7)	4409 (7.9)	11052 (5.8)	
**Facility location**				
** Midwest**	614 (29.5)	14132 (25.2)	46644 (24.6)	<0.001
** Northeast**	410 (19.7)	10411 (18.6)	37787 (20)	
** South**	716 (34.4)	20595 (36.8)	66519 (35.1)	
** West**	246 (11.8)	6451 (11.5)	27271 (14.4)	
** Unknown**	98 (4.7)	4409 (7.9)	11052 (5.8)	
**Clinical disease stage**				
** I**	639 (30.7)	25843 (46.1)	102257 (54)	<0.001
** II**	1215 (58.3)	23912 (42.7)	70846 (37.4)	
** III**	201 (9.6)	5579 (10)	14283 (7.5)	
** IV**	29 (1.4)	664 (1.2)	1887 (1)	
**Clinical T status**				
** cT0**	5 (0.2)	131 (0.2)	341 (0.2)	<0.001
** cT1**	674 (32.3)	28452 (50.8)	113113 (59.8)	
** cT2**	1026 (49.2)	21530 (38.4)	61145 (32.3)	
** cT3**	252 (12.1)	3702 (6.6)	10061 (5.3)	
** cT4**	127 (6.1)	2183 (3.9)	4613 (2.4)	
**Clinical N status**				
** cN0**	1787 (85.7)	43357 (77.4)	150890 (79.7)	<0.001
** cN1**	224 (10.7)	9659 (17.2)	30427 (16.1)	
** cN2**	51 (2.4)	1804 (3.2)	5414 (2.9)	
** cN3**	22 (1.1)	1178 (2.1)	2542 (1.3)	
**Pathologic stage**				
** 0**	45 (2.2)	4314 (7.7)	5361 (2.8)	<0.001
** 1**	582 (27.9)	24787 (44.3)	81680 (43.2)	
** 2**	1223 (58.7)	20790 (37.1)	72445 (38.3)	
** 3**	210 (10.1)	5675( 10.1)	28323 (15)	
** 4**	24 (1.2)	432 (0.8)	1464 (0.8)	
**Pathologic T status**				
** pTis**	7 (0.3)	432 (0.8)	1862 (1)	<0.001
** pT0**	42 (2)	4250 (7.6)	3972 (2.1)	
** pT1**	634 (30.4)	29055 (51.9)	106130 (56.1)	
** pT2**	1017 (48.8)	18530 (33.1)	63484 (33.5)	
** pT3**	290 (13.9)	2540 (4.5)	10588 (5.6)	
** pT4**	93 (4.5)	1097 (2)	2892 (1.5)	
** pTX**	1 (0)	94 (0.2)	345 (0.2)	
**Pathologic N status**				
** pN0**	1705 (81.9)	41587 (74.3)	113787 (60.2)	<0.001
** pN1**	284 (13.6)	9725 (17.4)	51483 (27.2)	
** pN2**	66 (3.2)	3031 (5.4)	16111 (8.5)	
** pN3**	27 (1.3)	1607 (2.9)	7685 (4.1)	
**Receptor grouping**				
** HR(+)/HER2(-)**	334 (16)	0 (0)	143687 (75.9)	<0.001
** Triple negative**	1604 (77)	55998 (100)	0 (0)	
** HER2(+)**	97 (4.7)	0 (0)	38726 (20.5)	
** Unknown**	49 (2.4)	0 (0)	6860 (3.6)	
**Tumor grade**				
** 1**	41 (2)	988 (1.8)	32015 (16.9)	<0.001
** 2**	237 (11.4)	9050 (16.2)	80590 (42.6)	
** 3**	1485 (71.3)	42930 (76.7)	65482 (34.6)	
** 4**	37 (1.8)	286 (0.5)	438 (0.2)	
** Unknown**	284 (13.6)	3030 (5.4)	11186 (5.9)	
**LVSI**				
** Not present**	1505 (72.2)	36400 (65)	118082 (62.4)	<0.001
** Present**	263 (12.6)	11091 (19.8)	45681 (24.1)	
** Unknown**	316 (15.2)	8507 (15.1)	25510 (13.4)	
**Type of surgery**				
** BCS**	876 (42)	29142 (52)	92022 (48.6)	<0.001
** Mastectomy**	1200 (57.6)	26561 (47.4)	95910 (50.7)	
** No surgery**	8 (0.4)	295 (0.5)	1341 (0.7)	
**Axillary surgery**				
** No surgery**	47 (2.3)	1034 (1.8)	4433 (2.3)	<.001
** SLND**	1353 (64.9)	34880 (62.3)	109718 (58)	
** ALND**	666 (32)	19229 (34.3)	73181 (38.7)	
** Unknown**	18 (0.9)	855 (1.5)	1941 (1)	
**Radiation therapy**				
** Yes**	1087 (52.2)	33938 (60.6)	109268 (57.7)	<.001
** No**	989 (47.5)	21860 (39)	79375 (41.9)	
** Unknown**	8 (0.4)	200 (0.4)	630 (0.3)	
**Radiation targets**				
** Breast/CW only**	803 (73.9)	24704 (72.8)	72410 (66.3)	<0.001
** Breast/CW + Regional nodes**	284 (26.1)	9234 (27.2)	36858 (33.7)	
**Chemotherapy**				
** Yes**	1571 (75.4)	44321 (79.1)	133965 (70.8)	<0.001
** No**	499 (23.9)	11195 (20)	53062 (28)	
** Unknown**	14 (0.7)	482 (0.9)	2246 (1.2)	
**Chemotherapy-to-surgery sequence**				
** Adjuvant**	1241 (59.5)	31519 (56.3)	116645 (61.6)	<0.001
** Neoadjuvant**	324 (15.5)	12581 (22.5)	16717 (8.8)	
** None**	519 (24.9)	11898 (21.2)	55911 (29.5)	
**Hormone therapy**				
** Yes**	220 (10.6)	1178 (2.1)	103906 (54.9)	<0.001
** No**	1798 (86.3)	53274 (95.1)	76820 (40.6)	
** Unknown**	66 (3.2)	1546 (2.8)	8547 (4.5)	
**Year of diagnosis**				
** 2010**	309 (14.8)	9605 (17.2)	36712 (19.4)	<0.001
** 2011**	393 (18.9)	11163 (19.9)	38815 (20.5)	
** 2012**	458 (22)	11205 (20)	39364 (20.8)	
** 2013**	463 (22.2)	11992 (21.4)	37764 (20)	
** 2014**	461 (22.1)	12033 (21.5)	36618 (19.3)	

Abbreviations: BC, breast cancer; ALND, axillary lymph node dissection; BCS, breast conserving surgery; CW, chest wall; HER2, human epidermal growth factor receptor; HR, hormone receptor; LVSI, lymphovascular invasion; SLND, sentinel lymph node dissection.

**Table 2:** Multivariable analysis of factors associated with overall survival for patients with metaplastic BC.

Factors Associated with Overall Survival in Metaplastic BC			
Variable (Reference)		Hazard Ratio (95% Confidence Interval)	*P* Value
Age	Per year increase	1.024 (1.015-1.033)	<0.0001
Lymphovascular Invasion (None)	Present	1.307 (1.017-1.679)	0.0364
Clinical N Status (cN0)	cN+	1.758 (1.328-2.326)	<0.0001
Clinical T Status (cT1)	cT0	2.226 (0.301-16.447)	<0.0001
	cT2	1.454 (1.102-1.918)	
	cT3	3.029 (2.183-4.204)	
	cT4	3.145 (2.124-4.657)	
Clinical M Status (cM0)	cM1	3.330 (2.020-5.488)	<0.0001
Axillary Surgery (SLND)	ALND	1.333 (1.065-1.670)	0.0247
	No Surgery	1.538 (0.884-2.675)	
Radiation (No)	Yes	0.709 (0.572-0.878)	0.0016
Chemotherapy (No)	Yes	0.579 (0.446-0.752)	<0.0001

Abbreviations: ALND, Axillary lymph node dissection; BC, breast cancer; SLND, sentinel lymph node dissection.
