# Supplementary material for: Outcomes after Treatment of Metaplastic Versus Other Breast Cancer Subtypes
Source: J Cancer. 2020 Jan 1;11(6):1341–50. doi: 10.7150/jca.40817 (PMC6995376; doi:10.7150/jca.40817)
Supplement: Supplementary file 1 — Supplementary table. [file jcav11p1341s1.pdf]

Supplemental Table 1. Multivariable analysis of factors associated with overall survival by cancer subtype

**Factors Associated with Overall Survival in Metaplastic BC**				**Factors Associated with Overall Survival in Triple-Negative BC**				**Factors Associated with Overall Survival in Other BC**			
**Variable (Reference)**		**Hazard Ratio (95% Confidence Interval)**	***P* Value**	**Variable (Reference)**		**Hazard Ratio (95% Confidence Interval)**	***P* Value**	**Variable (Reference)**		**Hazard Ratio (95% Confidence Interval)**	***P* Value**
**Age**	Per year increase	1.024 (1.015-1.033)	<0.0001	**Age**	Per year increase	1.013 (1.011-1.016)	<0.0001	**Age**	Per year increase	1.034 (1.032-1.036)	<0.0001
**Lymphovascular Invasion (None)**	Present	1.307 (1.017-1.679)	0.0364	**Lymphovascular Invasion (None)**	Present	1.984 (1.882-2.091)	<0.0001	**Lymphovascular Invasion (None)**	Present	1.439 (1.380-1.499)	<0.0001
**Clinical N Status (cN0)**	cN+	1.758 (1.328-2.326)	<0.0001	**Clinical N Status (cN0)**	cN+	1.670 (1.568-1.779)	<0.0001	**Clinical N Status (cN0)**	cN+	1.421 (1.355-1.491)	<0.0001
**Clinical T Status (cT1)**	cT0	2.226 (0.301-16.447)	<0.0001	**Clinical T Status (cT1)**	cT0	2.602 (1.433-4.725)	<0.0001	**Clinical T Status (cT1)**	cT0	1.555 (0.859-2.815)	<0.0001
	cT2	1.454 (1.102-1.918)			cT2	1.656 (1.562-1.756)			cT2	1.588 (1.521-1.657)	
	cT3	3.029 (2.183-4.204)			cT3	2.520 (2.307-2.753)			cT3	2.273 (2.114-2.444)	
	cT4	3.145 (2.124-4.657)			cT4	3.218 (2.917-3.550)			cT4	3.059 (2.821-3.317)	
**Clinical M Status (cM0)**	cM1	3.330 (2.020-5.488)	<0.0001	**Clinical M Status (cM0)**	cM1	3.599 (3.181-4.072)	<0.0001	**Clinical M Status (cM0)**	cM1	2.878 (2.608-3.176)	<0.0001
**Axillary Surgery (SLND)**	ALND	1.333 (1.065-1.670)	0.0247	**Axillary Surgery (SLND)**	ALND	1.341 (1.265-1.423)	<0.0001	**Axillary Surgery (SLND)**	ALND	1.282 (1.227-1.340)	<0.0001
	No Surgery	1.538 (0.884-2.675)			No Surgery	1.729 (1.482-2.017)			No Surgery	1.687 (1.548-1.839)	
**Radiation (No)**	Yes	0.709 (0.572-0.878)	0.0016	**Radiation (No)**	Yes	0.719 (0.678-0.763)	<0.0001	**Radiation (No)**	Yes	0.623 (0.596-0.651)	<0.0001

**Chemotherapy (No)**	Yes	0.579 (0.446-0.752)	<0.0001	**Chemotherapy (No)**	Yes	0.518 (0.486-0.552)	<0.0001	**Chemotherapy (No)**	Yes	0.486 (0.463-0.510)	<0.0001
				**Grade (1)**	2	1.366 (1.078-1.732)	<0.0001	**Grade (1)**	2	1.134 (1.073-1.199)	<0.0001
					3	1.708 (1.355-2.152)			3	1.687 (1.593-1.788)	
				**Comorbidity score (0)**	1	1.293 (1.215-1.376)	<0.0001	**Comorbidity score (0)**	1	1.429 (1.367-1.494)	<0.0001
					≥ 2	1.773 (1.610-1.952)			≥ 2	2.134 (1.997-2.281)	
				**Race (White)**	Black	1.088 (1.024-1.156)	<0.0001	**Race (White)**	Black	1.183 (1.119-1.251)	0.0002
					Other	0.787 (0.676-0.918)			Other	0.723 (0.646-0.810)	
				**Income Quartile (≥ $46,000)**	$35,000-$45,999	1.090 (1.027-1.157)	0.0044	**Income Quartile (≥ $46,000)**	$35,000-$45,999	1.100 (1.046-1.157)	0.0002
					$30,000-$34,999	1.114 (1.041-1.192)			$30,000-$34,999	1.134 (1.065-1.208)	
					<$30,000	1.038 (0.960-1.123)			<$30,000	1.144 (1.058-1.237)	
				**Insurance Status (Private Insurance)**	Medicare	1.323 (1.234-1.418)	<0.0001	**Insurance Status (Private Insurance)**	Medicare	1.356 (1.288-1.428)	<0.0001
					Medicaid	1.382 (1.269-1.505)			Medicaid	1.573 (1.463-1.692)	
					Other Government	0.984 (0.760-1.274)			Other Government	1.406 (1.182-1.672)	
					Not Insured	1.250 (1.075-1.453)			Not Insured	1.525 (1.348-1.725)	

	**Breast Surgery (BCS)**	Mastectomy	1.107 (1.039-1.180)	0.009	**Breast Surgery (BCS)**	Mastectomy	0.988 (0.944-1.034)	0.0004
		No Surgery	1.514 (1.097-2.089)			No Surgery	1.408 (1.180-1.681)	
					**No High School Quartile (<14%)**	14.0-19.9%	1.092 (1.037-1.149)	0.0002
						20.0-28.9%	1.086 (1.026-1.151)	
						≥ 29%	0.992 (0.922-1.067)	
					**Location (Metropolitan)**	Rural	1.014 (0.890-1.156)	0.002
						Urban	1.100 (1.043-1.160)	

Abbreviations: ALND, Axillary lymph node dissection; BC, breast cancer; BCS, breast-conserving surgery; OS, overall survival; SLND, sentinel lymph node dissection
